# Propofol, Sevoflurane, and Ketamine Induce a Reversible Increase in Delta-Gamma and Theta-Gamma Phase-Amplitude Coupling in Frontal Cortex of Rat

**DOI:** 10.3389/fnsys.2017.00041

**Published:** 2017-06-13

**Authors:** Dinesh Pal, Brian H. Silverstein, Lana Sharba, Duan Li, Viviane S. Hambrecht-Wiedbusch, Anthony G. Hudetz, George A. Mashour

**Affiliations:** ^1^Department of Anesthesiology, University of MichiganAnn Arbor, MI, United States; ^2^Center for Consciousness Science, University of MichiganAnn Arbor, MI, United States; ^3^Translational Neuroscience Program, Wayne State University School of MedicineDetroit, MI, United States; ^4^Neuroscience Graduate Program, University of MichiganAnn Arbor, MI, United States

**Keywords:** anesthesia, ketamine, phase-amplitude coupling, power spectrum, propofol, sevoflurane

## Abstract

Studies from human and non-human species have demonstrated a breakdown of functional corticocortical connectivity during general anesthesia induced by anesthetics with diverse molecular, neurophysiological, and pharmacological profiles. Recent studies have demonstrated that changes in long-range neural communication, and by corollary, functional connectivity, might be influenced by cross-frequency coupling (CFC) between the phase of slow oscillations and the amplitude of local fast oscillations. Phase-amplitude coupling (PAC) between slow oscillations and alpha rhythm during general anesthesia reveal distinct patterns depending on the anesthetic. In this study, we analyzed the effect of three clinically used anesthetics (propofol: *n* = 6, sevoflurane: *n* = 10, and ketamine: *n* = 8) with distinct molecular mechanisms on changes in PAC in the frontal cortex of rat. The loss of righting reflex was used as a surrogate for unconsciousness. PAC was calculated using the modulation index (MI) algorithm between delta (1–4 Hz), theta (4–10 Hz), low gamma (25–55 Hz), and high gamma (65–125 Hz) bands. A linear mixed model with fixed effects was used for statistical comparisons between waking, anesthetized, and post-anesthesia recovery epochs. All three anesthetics increased the coupling between delta and low gamma (*p* < 0.0001) as well as between theta and low gamma (*p* < 0.0001) oscillations, which returned to baseline waking levels during the post-anesthetic recovery period. In addition, a reversible reduction in high gamma power (*p* < 0.0001) was a consistent change during anesthesia induced by all three agents. The changes in delta-high gamma and theta-high gamma PAC as well as power spectral changes in delta, theta, and low gamma bandwidths did not show a uniform response across the three anesthetics. These results encourage the study of alternative PAC patterns as drug-invariant markers of general anesthesia in humans.

## Introduction

Loss of connected consciousness is a functional outcome common to general anesthetics with diverse molecular, pharmacological, and neurophysiological effects. Recent studies have found that a wide variety of general anesthetics disrupt intracortical network connectivity (Imas et al., 2005; Lee et al., 2009; Boveroux et al., 2010; Ferrarelli et al., 2010; Ku et al., 2011; Boly et al., 2012; Hudetz, 2012; Lewis et al., 2012; Casali et al., 2013; Jordan et al., 2013; Lee H. et al., 2013; Lee U. et al., 2013; Monti et al., 2013; Raz et al., 2014; Pal et al., 2015, 2016; Palanca et al., 2015; Bonhomme et al., 2016; Hudetz and Mashour, 2016; Ranft et al., 2016; Schroeder et al., 2016). Long-range slow oscillations have been shown to entrain local fast oscillations through cross-frequency coupling (CFC), which is likely to have a mechanistic influence on intracortical as well as cortical-subcortical connectivity (Canolty and Knight, 2010; Hyafil et al., 2015). Consistent with its potential ability to facilitate neural communication, CFC has been implicated in a wide variety of cognitive functions, including mnemonic processes (Canolty et al., 2006; Tort et al., 2008; Axmacher et al., 2010; Canolty and Knight, 2010; Lisman and Jensen, 2013; van Wingerden et al., 2014), sensory processing (Lakatos et al., 2008), input discrimination (Händel and Haarmeier, 2009), and motor planning and execution (Yanagisawa et al., 2012; Combrisson et al., 2016).

Phase-amplitude coupling (PAC), a form of CFC, has been demonstrated for numerous brain oscillations across species (Tort et al., 2009; Voytek et al., 2010; Scheffzük et al., 2011; López-Azcárate et al., 2013; Purdon et al., 2013; Blain-Moraes et al., 2014, 2015; van Wingerden et al., 2014; Berman et al., 2015; Takeuchi et al., 2015). In human subjects, studies exploring PAC between slow oscillations and the alpha rhythm as a function of anesthetic-induced unconsciousness demonstrated agent specific effects; propofol increased the coupling while sevoflurane and ketamine did not induce significant changes (Purdon et al., 2013; Blain-Moraes et al., 2014, 2015). In this rodent study, we tested the modulation of PAC in alternative frequencies by diverse anesthetics with the objective of identifying anesthetic-invariant patterns. Unlike the alpha rhythm in humans, delta-theta-gamma oscillations in rodents have been the focus of most investigations, including CFC analyses, and have been linked to cognitive functions as well as states of consciousness (Maloney et al., 1997; Tort et al., 2008, 2009; Hudetz et al., 2011; Scheffzük et al., 2011; Colgin, 2013; Lisman and Jensen, 2013; López-Azcárate et al., 2013; Pal et al., 2015, 2016). Therefore, in this study we investigated the effects of three clinically used anesthetics—propofol, sevoflurane, and ketamine—on PAC between delta (1–4 Hz), theta (4–10 Hz), low gamma (25–55 Hz), and high gamma (65–125 Hz) oscillations in the frontal cortex of rat. We demonstrate that, despite their distinct molecular and pharmacologic characteristics, the three anesthetics induce similar changes in delta-low gamma and theta-low gamma PAC during unconsciousness. These results motivate the study of delta/theta-low gamma PAC—or other unexplored CFC relationships—in humans as a potential anesthetic-invariant index to track states of consciousness in the clinical setting.

## Materials and Methods

### Data Acquisition

**Figure 1:**
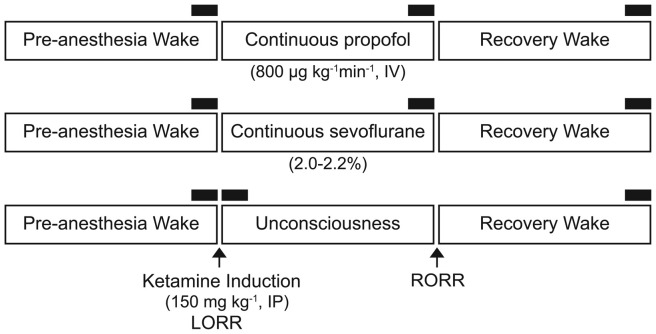
Schematics illustrating the experimental design to collect electroencephalogram (EEG) data before, during, and after the anesthetized state. Separate groups of rats were used for each anesthetic experiment. The black bars represent 750-s segments of EEG used for phase-amplitude coupling and power spectral density analysis. IP, intraperitoneal; IV, intravenous; LORR, loss of righting reflex; RORR, return of righting reflex.

We re-analyzed the electroencephalographic data sets collected for our recently published studies (Pal et al., 2015, 2016) to assess local PAC between delta, theta, and gamma bands before, during, and after propofol (*n* = 6), sevoflurane (*n* = 10), and ketamine (*n* = 8) anesthesia. Given the accessibility of the frontal area for electroencephalogram (EEG) recording and processing in clinical settings, we focused on EEG recorded from the frontal cortex (Bregma: anterior-posterior: +3.0 mm, medial-lateral: 2.5 mm) to enhance the translational relevance. Detailed surgical procedures and methodological approach are provided in our previous publications (Pal et al., 2015, 2016). In brief, all studies were conducted on adult male Sprague-Dawley rats (*n* = 24, 300–350 g, Charles River Laboratories Inc., Wilmington, MA, USA). The experimental procedures were approved by the Institutional Animal Care and Use Committee at the University of Michigan (Ann Arbor, MI, USA) and were in compliance with the Guide for the Care and Use of Laboratory Animals (8th Edition, The National Academies Press, Washington, DC, USA) as well as the ARRIVE guidelines (Kilkenny et al., 2010). Under surgical isoflurane anesthesia, the rats were instrumented with: (1) stainless steel screw electrodes to record monopolar cortical EEG; and (2) a guide tube in prefrontal cortex for microdialysis measurement of acetylcholine levels (not reported in this study), before, during, and after anesthetic-induced unconsciousness. A screw electrode over frontal sinus served as the reference for EEG recordings. Each rat received buprenorphine hydrochloride (Buprenex^®^, Reckitt Benckiser Pharmaceuticals Inc., Richmond, VA, USA) for pre-surgical (0.01 mg kg^−1^, s.c.) and post-surgical (0.03 mg kg^−1^, s.c., every 8–12 h for 24 h) analgesia as well as a single pre-surgical dose (20 mg kg^−1^, s.c.) of antibiotic cefazolin (West-Ward-Pharmaceutical Corp., Eatontown, NJ, USA). All rats were provided at least 7–10 days of post-surgical recovery during which they were conditioned to the recording chamber and recording cables. Microdialysis data from these studies were reported in our previous publications (Pal et al., 2015, 2016) and will not be presented here. Monopolar EEG, with reference to screw electrode over nasal sinus, was recorded between 0.1–300 Hz at a sampling rate of 1 kHz. The EEG signals were amplified (5000×) with a Grass Model 15 LT bipolar portable physiodata amplifier system (15A54 Quad Amplifier, Natus Neurology Inc., Warwick, RI, USA). A MP150 data acquisition unit along with Acq*knowledge* software (version 4.1.1, Biopac Systems, Inc., Goleta, CA, USA) was used for digitizing and storing the data. Figure 1 shows the experimental design for collecting EEG data before, during, and after anesthetic-induced unconsciousness. Loss of righting reflex was used as a surrogate for loss of consciousness. Propofol (800 μg kg^−1^ min^−1^) was delivered through a chronically implanted intravenous catheter in jugular vein while ketamine (150 mg kg^−1^ body wt.) was administered intraperitoneally. Sevoflurane (2.0%–2.2%) was delivered in a custom-made airtight round chamber that allowed EEG recordings. In order to hold the behavioral state constant, the rats in the propofol and sevoflurane groups were kept awake using gentle handling for 75 min prior to induction of anesthesia. Immediately after the completion of 75 min of pre-anesthesia EEG recording, the rats received either propofol or sevoflurane for 75 min, and the EEG data were recorded. Thereafter, the EEG data during the recovery waking state were collected for 75 min in the sevoflurane group. Given the differences in pharmacokinetics of sevoflurane and propofol, and to ensure complete recovery from the effects of intravenous propofol, the EEG data in the propofol group were recorded for an additional 75 min. Similar to propofol and sevoflurane groups, the rats in the ketamine group were kept awake using gentle handling for 40 min and thereafter received a single bolus dose of intraperitoneal ketamine. The EEG was not recorded between the ketamine injection and the onset of unconsciousness as assessed through the loss of righting reflex. The original study required collection of microdialysis samples because of which the data collection (EEG and microdialysis) resumed after 7 min of loss of righting reflex. Due to the individual variation in parenteral uptake of ketamine anesthetic and pharmacokinetics, the duration of anesthetized state varied in this group and therefore the EEG data during the anesthetized state were collected until the return of righting reflex. Thereafter, recovery wake EEG data were collected until the microdialysis levels of prefrontal acetylcholine returned to the pre-anesthetic wake levels. The EEG during the recovery waking state in the ketamine group was recorded in three out of eight rats.

### Data Analysis

#### Data Preprocessing

In order to obtain the most stable and representative data for the behavioral states, the EEG segments for data analysis were selected from last 750 s of wake, anesthetized, and recovery waking condition for both propofol and sevoflurane groups. In the ketamine group, the EEG segments for analysis were collected from the last 750 s during the waking state, the first 750 s after the ketamine induction and resumption of data collection, and the last 750 s in the recovery epoch. EEG data (750 s) corresponding to the freely moving pre-anesthesia baseline waking state, anesthetic-induced unconsciousness, and post-anesthetic recovery wakefulness were windowed into 30-s time bins. Motion artifacts were removed using a combination of EEGLAB (Delorme and Makeig, 2004) and visual inspection. Due to the sensitivity of PAC measures to sharp peak artifacts (Kramer et al., 2008), EEG data bins containing EMG interference and/or peaks beyond ±25 μV were excluded.

#### Modulation Index

To compute PAC, we employed the modulation index (MI) method (Canolty et al., 2006) using the PAC Toolbox (Onslow et al., 2011) for MATLAB (MathWorks Inc., Natick, MA, USA). The MI was selected over other PAC measures (e.g., cross-frequency coherence or envelope-to-signal correlation) due to its relative robustness for detecting PAC despite low amplitude in the high frequency signal and the possibility of high background noise (Onslow et al., 2011).

We employed wavelet convolution (Morlet wavelet, width = 7) and used filt_signalsWAV.m function in the PAC Toolbox (Onslow et al., 2011) to bandpass filter the data into 2 Hz bins for the phase signals and 10 Hz bins for the amplitude signals. Wavelet convolution returns an analytic signal, $s_{\text{filt}}(k)=A(k)e^{i\theta (k)}$ for each filtered waveform, where *A*(*k*) is the instantaneous amplitude, *θ* (*k*) is the instantaneous phase, *k* = 1,2, … , *n*, and *n* is the number of data points in a 30-s window. The phase term of the low frequency signal, *θ*_LF_(*k*), is extracted and combined with the amplitude term of the high frequency signal, *A*_HF_(*k*), to create a third composite signal, $z(k)=A_{\text{HF}}(k)e^{i\theta_{\text{LF}}(k)}$. The raw MI is then equal to the absolute value of the mean vector length of this complex composite signal, $MI_{\text{raw}}=\frac{1}{n}\sum_{k=1}^{n}\left|z(k)\right|$. This approach was applied to each frequency pair in each 30-s window, resulting in *MI*_raw_. In order to define the statistically significant level of PAC, the filtered time series were temporally shuffled using the random insertion approach as implemented in the PAC Toolbox (Onslow et al., 2011). This process was conducted 200 times for each 30-s time window. PAC was computed as described above for each shuffled time series to create a surrogate data set of spurious PAC values, *MI*_spur_. Values of *MI*_raw_ were then compared to the mean, *μ*_spur_ and standard deviation, *σ*_spur_, of the *MI*_spur_ distribution to create normalized PAC values equivalent to *z*-scores, $MI_{norm}=\frac{MI_{raw}-\mu_{\text{spur}}}{\sigma_{\text{spur}}}$ (Canolty et al., 2006). A threshold of *MI*_norm_ > 1.645 (or *α* < 0.05) was set for determining PAC significance; the values that surpassed this threshold were used for further analysis. Modulograms for each state—baseline waking, anesthetized, and recovery waking—were then generated by averaging significant values of *MI*_norm_ across analysis windows within each state. PAC for specific frequency band pairs of interest was calculated by averaging thresholded *MI*_norm_ values across frequency bins (specifically, delta: 1–4 Hz, theta: 4–10 Hz, low gamma: 25–55 Hz, and high gamma: 65–125) and analysis windows. To determine the phase of low-frequency signal at which observed PAC occurred, the coupling phase in each analysis window, *θ*_LF_, was averaged within the delta and theta bands and plotted against corresponding values of *MI*_norm_.

#### Power Spectral Density

In order to confirm the independence of PAC from the spectral changes, as was demonstrated in a recent study (Mukamel et al., 2014), we calculated power spectral density (PSD) in the bandwidths analyzed for PAC. As was done with the PAC calculation, PSD was calculated in 30-s bins. Absolute PSD in each of the frequency bands of interest (delta: 0.5–4 Hz, theta: 4–10 Hz, low gamma: 25–55 Hz, and high gamma: 65–125 Hz) was calculated based on the short-time Fourier transform using the “spectrogram.m” function in the MATLAB Signal Processing Toolbox (Borjigin et al., 2013; Pal et al., 2015, 2016) as well as Welch’s method (pwelch.m function in Matlab Signal Processing Toolbox). For Welch’s method, the 30-s epoch was divided into 10-s sub-epochs with 80% overlap, and a modified periodogram was computed for each sub-epoch using a Hamming window. All the resulting periodograms were averaged to compute the absolute spectral estimate. Relative power was calculated by dividing the power in each 0.5 Hz frequency bin by the total power in the band (0.5–300 Hz), and then summing within a particular frequency band. The PSD data for the ketamine group was reported in a previous publication in 10-s bins from our laboratory (Pal et al., 2015) but was recalculated for this study.

### Statistical Analysis

Statistical analysis was conducted in consultation with the *Consulting for Statistics, Computing and Analytics Research* unit at the University of Michigan, Ann Arbor, MI, USA. A linear mixed model with fixed effects was used for comparison of the MI (delta-low gamma, theta-low gamma, delta-high gamma, and theta-high gamma) and relative PSD (delta, theta, low gamma, and high gamma) between the following conditions: (1) pre-anesthesia baseline waking state, (2) anesthetic-induced unconsciousness (propofol, sevoflurane, ketamine), and (3) post-anesthetic recovery wakefulness. The data are reported as mean ± standard error of the mean (SEM) along with 95% confidence intervals (CI). The statistical analyses were conducted using the programming and statistical language R (R Core Team, 2016).

## Results

### Phase-Amplitude Coupling before, during, and after Propofol, Sevoflurane, and Ketamine-Induced Unconsciousness

**Figure 2:**
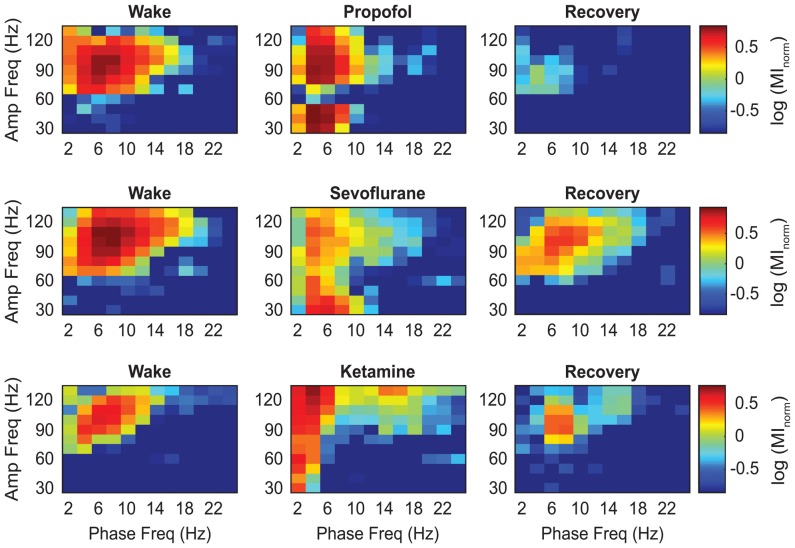
Representative heat maps showing the changes in coupling between the phase of low frequency and the amplitude of high frequency oscillations. The colors represent the log-transformed modulation strength as illustrated in the vertical scale bar. Warmer colors indicate high PAC while cooler colors indicate low PAC. Amp, amplitude; Freq, frequency; MI_norm_, normalized modulation index.

**Figure 3:**
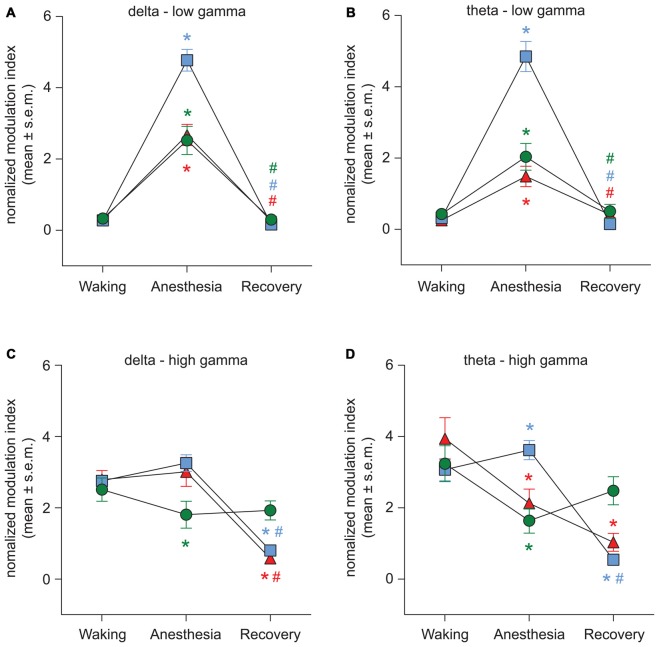
Effect of propofol, sevoflurane, and ketamine on PAC in delta-low gamma **(A)**, theta-low gamma **(B)**, delta-high gamma **(C)**, and theta-high gamma **(D)** frequency pairs. Significance symbols denote a statistical difference at an alpha of *p* < 0.05. The actual *p* values are reported in the text in the results section. *significant as compared to waking, ^#^significant as compared to anesthetic-induced unconsciousness, blue squares: propofol group, green circles: sevoflurane group, red triangles: ketamine group. Significance symbols are color-coded and match the symbol colors for the experimental groups. For some of the data points and groups, the error bars are shorter than the height of the symbol and therefore are not visible. SEM, standard error of the mean.

The representative modulograms in Figure 2 illustrate the PAC before, during, and after anesthetic-induced unconsciousness. An increase in delta-theta and theta-low gamma coupling can be observed across all three anesthetics. After averaging within target frequency bands across all rats, statistical analysis showed that, compared to the pre-anesthesia waking state, all three anesthetics induced a significant increase in delta-low gamma (*p* < 0.0001 for propofol, sevoflurane, and ketamine) and theta-low gamma (*p* < 0.0001 for propofol, sevoflurane, and ketamine) PAC (Figures 3A,B, Tables 1–3). There was no significant difference in delta-high gamma PAC during propofol (*p* = 0.06), and ketamine (*p* = 0.6)-induced unconsciousness as compared to the waking state while delta-high gamma PAC showed a significant reduction during sevoflurane-induced (*p* = 0.03) unconsciousness (Figure 3C, Tables 1–3). Theta-high gamma PAC showed a significant increase during propofol-induced unconsciousness (*p* = 0.03 vs. waking) while there was a significant decrease in theta-high gamma PAC during sevoflurane (*p* = 0.004 vs. waking) and ketamine (*p* = 0.004 vs. waking) anesthesia (Figure 3D, Tables 1–3).

**Table 1:** Phase-amplitude coupling and power spectral density before, during, and after propofol-induced unconsciousness.

		Phase-Amplitude Coupling
Frequency pair (Hz)	Wake	Propofol	Recovery wake	*F* statistic *p* value
Delta-low gamma	0.28 ± 0.044	4.8 ± 0.3	0.16 ± 0.015	*F*_(2,15)_ = 218.9
	(0.16–0.39)	(4.0–5.6)	(0.12–0.20)	*p < 0.0001
Theta-low gamma	0.32 ± 0.06	4.8 ± 0.42	0.15 ± 0.026	*F*_(2,10)_ = 124.3
	(0.16–0.47)	(3.8–5.9)	(0.08–0.21)	*p < 0.0001
Delta-high gamma	2.8 ± 0.16	3.3 ± 0.23	0.8 ± 0.15	*F*_(2,15)_ = 49.4
	(2.3–3.2)	(2.7–3.9)	(0.42–1.2)	*p < 0.0001
Theta-high gamma	3.1 ± 0.31	3.6 ± 0.27	0.54 ± 0.14	*F*_(2,10)_ = 81.02
	(2.3–3.9)	(2.9–4.3)	(0.19–0.90)	*p < 0.0001
		**Power Spectral Density**
**Frequency band (Hz)**	**Wake**	**Propofol**	**Recovery wake**	***F* statistic *p* value**
Delta	56 ± 4.6	55 ± 3.1	50 ± 1.9	*F*_(2,15)_ = 0.77
	(44–67)	(47–63)	(45–55)	*p* = 0.5
Theta	22 ± 3.4	25 ± 1.2	15 ± 1.2	*F*_(2,10)_ = 7.4
	(14–31)	(22–28)	(12–18)	*p = 0.01
Low gamma	1.3 ± 0.12	1.2 ± 0.064	3.1 ± 0.83	*F*_(2,15)_ = 5.2
	(0.97–1.6)	(1.0–1.3)	(1.0–5.3)	*p = 0.02
High gamma	0.35 ± 0.04	0.01 ± 0.001	0.39 ± 0.041	*F*_(2,15)_ = 39.0
	(0.25–0.46)	(0.01–0.017)	(0.28–0.49)	*p < 0.0001

*Data are shown as mean ± standard error of the mean and 95% confidence intervals in parenthesis. Asterisk indicates significant difference*.

**Table 2:** Phase-amplitude coupling and power spectral density before, during, and after sevoflurane-induced unconsciousness.

		Phase-Amplitude Coupling
Frequency pair (Hz)	Wake	Sevoflurane	Recovery wake	*F* statistic *p* value
Delta-low gamma	0.33 ± 0.064	2.5 ± 0.4	0.3 ± 0.091	*F*_(2,18)_ = 30.9
	(0.19–0.47)	(1.6–3.4)	(0.092–0.51)	*p < 0.0001
Theta-low gamma	0.43 ± 0.081	2.0 ± 0.38	0.5 ± 0.2	*F*_(2,27)_ = 13.2
	(0.24–0.61)	(1.2–2.9)	(0.062–0.95)	*p < 0.0001
Delta-high gamma	2.5 ± 0.33	1.8 ± 0.38	1.9 ± 0.27	*F*_(2,18)_ = 2.7
	(1.8–3.2)	(0.95–2.7)	(1.3–2.5)	*p* = 0.1
Theta-high gamma	3.2 ± 0.5	1.6 ± 0.35	2.5 ± 0.39	*F*_(2,18)_ = 4.1
	(2.1–4.4)	(0.84–2.4)	(1.6–3.4)	*p = 0.03
		**Power Spectral Density**
**Frequency band (Hz)**	**Wake**	**Sevoflurane**	**Recovery wake**	***F* statistic *p* value**
Delta	54 ± 3.2	64 ± 3.6	49 ± 3.3	*F*_(2,18)_ = 7.2
	(47–61)	(56–72)	(41–56)	*p = 0.005
Theta	22 ± 2.6	14 ± 1.9	21 ± 2.1	*F*_(2,18)_ = 1.9
	(16–28)	(10–18)	(16–26)	*p* = 0.2
Low gamma	1.1 ± 0.093	1.4 ± 0.3	1.5 ± 0.17	*F*_(2,18)_ = 1.9
	(0.89–1.3)	(0.73–2.1)	(1.1–1.9)	*p* = 0.2
High gamma	0.28 ± 0.042	0.029 ± 0.005	0.37 ± 0.07	*F*_(2,27)_ = 15.5
	(0.18–0.37)	(0.018–0.039)	(0.22–0.52)	*p < 0.0001

*Data are shown as mean ± standard error of the mean and 95% confidence intervals in parenthesis. Asterisk indicates significant difference*.

**Table 3:** Phase-amplitude coupling and power spectral density before, during, and after ketamine-induced unconsciousness.

		Phase-Amplitude Coupling
Frequency pair (Hz)	Wake	Ketamine	Recovery wake	*F* statistic *p* value
Delta-low gamma	0.3 ± 0.045	2.7 ± 0.3	0.26 ± 0.042	*F*_(2,16)_ = 41.3
	(0.19–0.4)	(2–3.4)	(0.079–0.44)	*p < 0.0001
Theta-low gamma	0.25 ± 0.03	1.5 ± 0.29	0.41 ± 0.079	*F*_(2,10.5)_ = 12.5
	(0.18–0.32)	(0.81–2.2)	(0.071–0.75)	*p = 0.002
Delta-high gamma	2.8 ± 0.25	3.0 ± 0.41	0.58 ± 0.16	*F*_(2,16)_ = 8.5
	(2.2–3.4)	(2.0–4.0)	(−0.094–1.3)	*p = 0.003
Theta-high gamma	3.9 ± 0.59	2.1 ± 0.4	1.0 ± 0.25	*F*_(2,8.8)_ = 6.9
	(2.5–5.3)	(1.2–3.1)	(−0.045–2.1)	*p = 0.02
		**Power Spectral Density**
**Frequency band (Hz)**	**Wake**	**Ketamine**	**Recovery wake**	***F* statistic *p* value**
Delta	58 ± 2.6	60 ± 3.4	57 ± 2.8	*F*_(2,9.3)_ = 0.25
	(52–64)	(52–68)	(45–69)	*p* = 0.8
Theta	24 ± 2.3	21 ± 2.6	22 ± 3.1	*F*_(2,8.4)_ = 0.4
	(19–30)	(15–28)	(8.2–35)	*p* = 0.7
Low gamma	1.2 ± 0.088	2.0 ± 0.55	1.6 ± 0.22	*F*_(2,16)_ = 1.1
	(0.98–1.4)	(0.67–3.3)	(0.63–2.5)	*p* = 0.4
High gamma	0.56 ± 0.089	0.12 ± 0.03	0.39 ± 0.1	*F*_(2,16)_ = 11.2
	(0.35–0.77)	(0.048–0.19)	(−0.037–0.83)	*p = 0.0009

*Data are shown as mean ± standard error of the mean and 95% confidence intervals in parenthesis. Asterisk indicates significant difference*.

**Figure 4:**
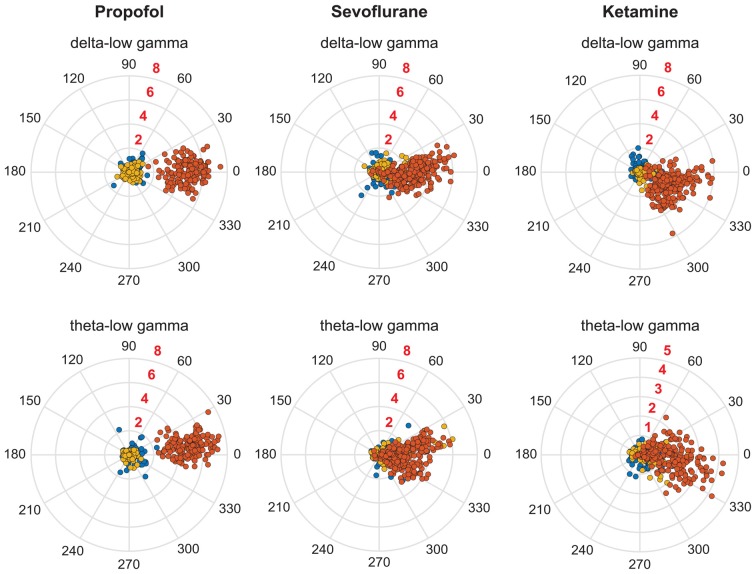
Coupling phase distribution of PAC during pre-anesthesia waking state (blue dots), anesthetic-induced unconsciousness (orange dots), and post-anesthetic recovery epoch (golden dots). Each dot represents the PAC in a 30-s window from one rat; all analyzed rats and windows are displayed. The numbers in black around the periphery denote the phase angle. The numbers in red embedded in each consecutive circle denote the strength of PAC.

The recovery waking state after propofol, sevoflurane, and ketamine-induced unconsciousness was characterized by a significant decrease in PAC in delta-low gamma (*p* < 0.0001 for propofol, sevoflurane, and ketamine) and theta-low gamma (propofol: *p* < 0.0001, sevoflurane: *p* < 0.0001, and ketamine: *p* = 0.003; Figures 3A,B, Tables 1–3). There was no significant difference between waking and post-anesthetic recovery for delta-low gamma (propofol: *p* = 0.6, sevoflurane: *p* = 0.9, and ketamine: *p* = 0.9) and theta-low gamma (propofol: *p* = 0.6, sevoflurane: *p* = 0.8, and ketamine: *p* = 0.6) PAC (Figures 3A,B, Tables 1–3). The post-propofol recovery epoch was also marked by a significant reduction in delta-high gamma (*p* < 0.0001 vs. waking, *p* < 0.0001 vs. unconscious state) and theta-high gamma (*p* < 0.0001 vs. waking, and *p* < 0.0001 vs. unconscious state) PAC (Figures 3C,D, Table 1). PAC in delta-high gamma and theta-high gamma did not show any significant differences during post-sevoflurane recovery period as compared to either the waking or unconscious state (delta-high gamma, *p* = 0.07 vs. waking, and *p* = 0.7 vs. unconscious state; theta-high gamma: *p* = 0.2 vs. waking, and *p* = 0.1 vs. unconscious state; Figures 3C,D, Table 2). Similarly, while there was no significant change in PAC in delta-high gamma between the waking state and ketamine anesthesia (*p* = 0.6), delta-high gamma PAC remained significantly lower during post-ketamine recovery (*p* = 0.0003 vs. waking, and *p* < 0.0001 vs. unconscious state; Figure 3C, Table 3). Theta-high gamma PAC was not significantly different between ketamine-induced unconsciousness and post-ketamine recovery epoch (*p* = 0.3) but was significantly lower as compared to the waking state (*p* = 0.001; Figure 3D, Table 3). In order to understand the phase preference of different anesthetics in delta- and theta-low gamma PAC, we plotted values of *MI*_norm_ in each analysis window against the phase of the low frequency component, *θ*_LF_ (Figure 4). Across all three anesthetic groups, during the waking and recovery state, delta- and theta-low gamma pairs shows low PAC and no clear phase preference. During the anesthetized state, PAC increased and shows a strong narrow distribution centered on zero phase, which is “peak-max” coupling as defined by Purdon and colleagues (Purdon et al., 2013).

### Power Spectral Density before, during, and after Propofol, Sevoflurane, and Ketamine-Induced Unconsciousness

**Figure 5:**
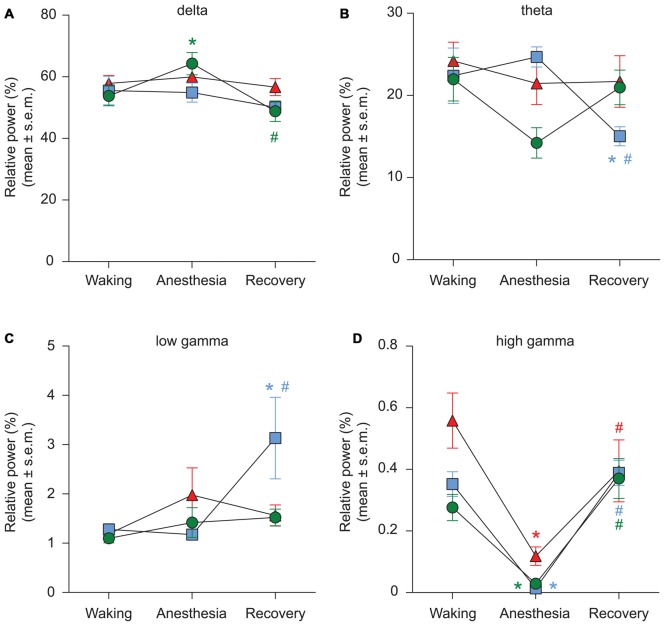
Effect of propofol, sevoflurane, and ketamine on relative PSD in delta **(A)**, theta **(B)**, low gamma **(C)**, and high gamma **(D)** bandwidths. Significance symbols denote a statistical difference at an alpha of *p* < 0.05. The actual *p* values are reported in the text in the results section. *significant as compared to waking, ^#^significant as compared to anesthetic-induced unconsciousness, blue squares: propofol group, green circles: sevoflurane group, red triangles: ketamine group. Significance symbols are color coded and match the symbol colors for the experimental groups. For some of the data points and groups, the error bars are shorter than the height of the symbol and therefore are not visible. SEM, standard error of the mean.

We used two different methods—short-time Fourier transform and Welch’s method—to analyze changes in spectral power. Because both methods showed similar changes in relative spectral power, we report only the results from analysis based on short-time Fourier transform. The results showed that neither propofol (*p* = 0.9) nor ketamine (*p* = 0.6) produced a significant change in delta power while there was a significant increase (*p* = 0.01) in delta power during sevoflurane-induced unconsciousness (Figure 5A, Tables 1–3). Even though relative power changes in delta were found to be variable across anesthetics, all three anesthetics produced an increase in absolute delta power (data not shown). Theta and low gamma power did not show any change from the waking state during propofol (theta: *p* = 0.4, low gamma: *p* = 0.9), sevoflurane (theta: *p* = 0.06, low gamma: *p* = 0.2), or ketamine-induced unconsciousness (theta: *p* = 0.4, low gamma: *p* = 0.1; Figures 5B,C, Tables 1–3). The delta power during post-propofol recovery period was not significantly different from that observed during either waking (*p* = 0.3) or propofol-induced unconsciousness (*p* = 0.3; Figure 5A, Table 1) while delta power during the post-sevoflurane epoch showed a significant decrease (*p* = 0.0002 vs. unconscious state) and reverted back to the pre-anesthesia baseline waking levels (*p* = 0.2 vs. unconscious state; Figure 5A, Table 2). Post-propofol recovery was also characterized by a significant decrease in theta power as compared to both pre-anesthesia waking (*p* = 0.005) and the unconscious state (*p* = 0.0002). In contrast, there was a significant increase in low gamma power during the post-propofol period as compared to both pre-anesthesia waking (*p* = 0.007) and propofol-induced unconsciousness (*p* = 0.004; Figures 5B,C, Table 1). Recovery from ketamine anesthesia was marked by the absence of any statistically significant change in delta (*p* = 0.8 vs. waking, and *p* = 0.5 vs. unconscious state), theta (*p* = 0.6 vs. waking, and *p* = 1.0 vs. unconscious state), and low gamma (*p* = 0.6 vs. waking, and *p* = 0.6 vs. unconscious state) bandwidths (Figures 5A–C, Table 3).

The most consistent PSD change across three anesthetics was observed in the high gamma bandwidth. Compared to the waking state, high gamma power showed a significant decrease during propofol (*p* < 0.0001), sevoflurane (*p* < 0.0001), and ketamine (*p* < 0.0001)-induced unconsciousness (Figure 5D, Tables 1–3). Furthermore, the high gamma power during recovery from all three anesthetics showed a significant increase and recovered back to the baseline waking levels (*p* < 0.0001 vs. propofol and sevoflurane; *p* = 0.03 vs. ketamine-induced unconsciousness); there was no significant difference in high gamma power between wakefulness and recovery epoch (propofol: *p* = 0.4, sevoflurane: *p* = 0.1, ketamine: *p* = 0.2; Figure 5D, Tables 1–3).

## Discussion

The main finding of this study is that the increase in delta-low gamma and theta-low gamma PAC is a common neurophysiological feature across three clinically-used anesthetics in the frontal cortex of rat. These anesthetic agents are characterized by distinct pharmacological and mechanistic profiles with respect to their receptor specificity: propofol is known to act primarily through facilitation of GABA neurotransmission while ketamine and sevoflurane exhibit a more diversified receptor target profile that includes both GABAergic and non-GABAergic receptors as well as various ion channels (Alkire et al., 2008; Drexler et al., 2011; Li and Vlisides, 2016; Wang et al., 2017). In addition, these anesthetic agents show differences in regional brain blood flow, metabolism, and functional connectivity (Bonhomme et al., 2011; Hudetz, 2012). Despite these differences, recent studies from our laboratory demonstrated a breakdown of frontal-parietal functional connectivity as a correlate of propofol, sevoflurane, and ketamine anesthesia across three different species—human, macaque, and rat (Lee U. et al., 2013; Pal et al., 2016; Schroeder et al., 2016). Thus, the present data lend further support to the hypothesis that there may be common neurophysiological markers or mechanisms of anesthetic-induced unconsciousness (Hudetz and Mashour, 2016).

Although CFC has been proposed as a mechanistic link between distinct neuronal assemblies that enables information transfer across multiple temporal domains (Canolty and Knight, 2010), a causal relationship between PAC and cognitive-behavioral functions has yet to be demonstrated. However, similar to the cross frequency interactions observed between delta-theta-gamma bands in the current study, it has previously been shown that delta phase modulates theta amplitude, and theta phase modulates gamma amplitude (Lakatos et al., 2005). Slow frequency phase modulation of high frequency amplitude has also been demonstrated across sensory modalities, brain regions, and species (Canolty et al., 2006; Lakatos et al., 2008; Tort et al., 2008, 2009; Händel and Haarmeier, 2009; Axmacher et al., 2010; Canolty and Knight, 2010; Voytek et al., 2010; Scheffzük et al., 2011; Yanagisawa et al., 2012; Lisman and Jensen, 2013; López-Azcárate et al., 2013; Purdon et al., 2013; Blain-Moraes et al., 2014, 2015; van Wingerden et al., 2014; Berman et al., 2015; Takeuchi et al., 2015; Combrisson et al., 2016), which considered together suggest that PAC may indeed serve a mechanistic role in local as well as inter-regional information transfer. Furthermore, our findings have translational potential because the anesthetic-invariant PAC pattern identified in this study is: (1) in frontal cortex, which is more clinically relevant because it can be assessed through a clinically-accessible site of recording, and (2) in bandwidths that can be measured using scalp EEG in humans. A study conducted on human volunteers found that propofol-induced unconsciousness was associated with an increase in PAC between slow oscillations and the alpha rhythm in the form of peak-max coupling (Purdon et al., 2013). However, similar studies conducted in human volunteers and surgical patients did not find frontal PAC between slow oscillations and the alpha rhythm either with sevoflurane- or ketamine-induced unconsciousness (Blain-Moraes et al., 2014, 2015). Considering the variable response of slow oscillations-alpha PAC across these anesthetics, which is likely a reflection of drug pharmacology, the results from the current study provide further motivation for exploration of PAC in delta-theta-gamma bandwidths, or other unexplored CFC relationships, in human subjects. However, it is important to note that in addition to gross differences in cortical anatomy, rodents and humans also show significant differences in EEG. The most prominent brain oscillation in human and higher species is the alpha rhythm, which does not appear to have the same functional equivalence or significance in rodents. In rodents, theta oscillations occupy the same prominence as alpha rhythm in humans, and have been shown to be associated with a variety of functions, including learning, memory, cognition, and states of consciousness (Maloney et al., 1997; Canolty et al., 2006; Tort et al., 2008, 2009; Hudetz et al., 2011; Scheffzük et al., 2011; Colgin, 2013; Lisman and Jensen, 2013; Pal et al., 2015, 2016). On the contrary, the existence and functional role of theta rhythm in humans has been a topic of debate although cumulative evidence from recent studies suggests the existence of slower theta oscillations, which are analogous to the typical theta frequency rhythm in rodents (reviewed in Jacobs, 2013). Furthermore, a direct functional link between hippocampus and prefrontal cortex in rodents has been demonstrated (Siapas et al., 2005; Sirota et al., 2008; Jacobs, 2013) that could be driving the increase in delta- and theta-gamma PAC in the frontal cortex. Despite the clear species-specific differences, the results from our study, along with relative ease of recording frontal EEG in humans, encourages further investigation of PAC changes in humans in the delta- and theta-low gamma frequency bands.

Phase-phase coupling, such as the SynchFastSlow component of bispectral analysis, has also been explored during general anesthesia (Nunes et al., 2012; Li et al., 2013). However, phase-phase coupling has been shown to be vulnerable to power spectral changes and spurious coupling (Miller et al., 2004; Scheffer-Teixeira and Tort, 2016) whereas changes in PAC were shown to be dissociated from power spectral changes (Mukamel et al., 2014). The dissociation between changes in PAC and spectral power was also evident in our results. Although all three anesthetics induced a significant increase in delta-low gamma and theta-low gamma PAC, the power in delta (except for sevoflurane), theta, and low gamma bands during the state of anesthesia did not show a significant difference from waking. However, there was a uniform increase in absolute delta and theta power (except with sevoflurane) across anesthetics while only ketamine induced an increase in low gamma power (data not shown). In contrast, high gamma power showed a significant and consistent decrease during unconsciousness induced by all three anesthetics. This is in agreement with previous studies showing that high-frequency gamma power decreases with propofol-induced unconsciousness in humans (Breshears et al., 2010), and with propofol (Reed and Plourde, 2015) and isoflurane (Hudetz et al., 2011; Plourde et al., 2016)—induced unconsciousness in rats. Increase in frontal alpha power during anesthesia (known as anteriorization) was first reported in Java monkeys (Tinker et al., 1977) and is a well-known phenomenon in humans (Purdon et al., 2013). However, ketamine does not produce similar changes in frontal alpha power (Blain-Moraes et al., 2014) and sevoflurane only appears to induce anteriorization at doses beyond what is required for loss of consciousness (Gugino et al., 2001; Akeju et al., 2014; Blain-Moraes et al., 2015; Kaskinoro et al., 2015) suggesting that changes in alpha power represent a drug effect rather than a state correlate.

Collectively, the PSD findings suggest that the changes in EEG spectral power cannot be generalized across anesthetic agents as potential indicators of the states of consciousness. The only exception is the changes in high gamma, which, however, are of limited clinical utility because of the technical difficulty of recording high-frequency oscillations with scalp EEG. Breakdown in frontal-parietal functional connectivity is another potential drug-invariant marker of general anesthesia but presents a practical difficulty because of the inconvenience of electrophysiological recording in more posterior areas during clinical care. Of note, our findings with PAC between delta/theta- low gamma correlate with changes observed in high-frequency oscillations and frontal-parietal connectivity. Unlike those measures, PAC in these bandwidths can be easily quantified through frontal EEG—already used for intraoperative monitoring—and is therefore feasible for translational/clinical application.

## Limitations

A major limitation of this study is that we did not investigate the PAC changes during induction and emergence from anesthesia. Transition into and out of anesthetic-induced unconsciousness in rodents is particularly difficult to characterize because it is assessed based on the loss of righting reflex, which is an indirect measure of unconsciousness and its determination involves frequent tactile stimulation that introduces EEG artifacts and interferes with the induction/emergence process itself. Investigation of induction/emergence processes in human subjects is likely to offer more reliable and robust data. Furthermore, it is possible that the bandwidths studied in rodents are of less relevance to state transitions and the anesthetized state in humans. Again, translation to human studies will be required to confirm the clinical significance of our findings.

In addition, a few methodological caveats should be considered when interpreting the PAC results described here. First, our results are limited in their ability to provide a mechanistic explanation for the PAC changes associated with anesthetic-induced unconsciousness. Fluctuations in PAC can be caused by the appearance or disappearance of non-linearities in the signal that are independent of neural coordination (Aru et al., 2015). Regardless of the underlying mechanisms, our results do suggest a correlative relationship between delta-low gamma and theta-low gamma coupling and anesthetic-induced unconsciousness, independently of anesthetic pharmacology. Second, the measure of MI used here has been criticized for its potential relationship to the power of the amplitude signal. However, when the normalized MI is calculated, as was done here, the magnitude of PAC is not dependent on amplitude signal power (Canolty et al., 2006; Tort et al., 2010).

In conclusion, PAC between delta/theta and low gamma oscillations is a drug-invariant marker of general anesthesia in rats that could be of potential translational value for intraoperative monitoring and clinical applications.

## Author Contributions

DP, BHS, and GAM designed the study. DP and VSH collected the data. DP, BHS, LS, and DL analyzed the data. DP, BHS, LS, DL, VSH, AGH and GAM interpreted the data and wrote the manuscript. All authors approved the final version of the manuscript.

## Conflict of Interest Statement

The authors declare that the research was conducted in the absence of any commercial or financial relationships that could be construed as a potential conflict of interest.
